# Mid-Frequency Hearing Loss Is Characteristic Clinical Feature of *OTOA*-Associated Hearing Loss

**DOI:** 10.3390/genes10090715

**Published:** 2019-09-16

**Authors:** Kenjiro Sugiyama, Hideaki Moteki, Shin-ichiro Kitajiri, Tomohiro Kitano, Shin-ya Nishio, Tomomi Yamaguchi, Keiko Wakui, Satoko Abe, Akiko Ozaki, Remi Motegi, Hirooki Matsui, Masato Teraoka, Yumiko Kobayashi, Tomoki Kosho, Shin-ichi Usami

**Affiliations:** 1 Department of Otorhinolaryngology, School of Medicine, Shinshu University, 3-1-1 Asahi, Matsumoto, Nagano 390-8621, Japan; ksugiyama@shinshu-u.ac.jp (K.S.); moteki@shinshu-u.ac.jp (H.M.); tomokitano@shinshu-u.ac.jp (T.K.);; 2 Department of Hearing Implant Sciences, Shinshu University School of Medicine, 3-1-1 Asahi, Matsumoto, Nagano 390-8621, Japan; 3 Department of Medical Genetics, Shinshu University School of Medicine, 3-1-1 Asahi, Matsumoto, Nagano 390-8621, Japan; t_yamaguchi@shinshu-u.ac.jp (T.Y.); kwakui@shinshu-u.ac.jp (K.W.); ktomoki@shinshu-u.ac.jp (T.K.); 4 Center for Medical Genetics, Shinshu University Hospital, Matsumoto Nagano 390-8621, Japan; 5 Department of Otorhinolaryngology, Toranomon Hospital, 2-2-2 Toranomon, Minato-ku, Tokyo 105-8470, Japan; abe3387@ybb.ne.jp; 6 Department of Otorhinolaryngology-Head and Neck Surgery, Osaka Medical College, 2-7 Daigaku-machi, Takatsuki, Osaka 569-8686, Japan; oto108@osaka-med.ac.jp; 7 Department of Otorhinolaryngology, Juntendo University, 2-1-1 Hongo, Bunkyo-ku 113-8421, Japan; 8 Department of Otolaryngology, Head and Neck Surgery, Yamagata University Faculty of Medicine, 2-2-2 Iida-Nishi, Yamagata-shi, Yamagata 990-9585, Japan; matsuihirooki@gmail.com; 9 Department of Otolaryngology, Head and Neck Surgery, Ehime University Graduate School of Medicine, 454 Shitsukawa, Toon, Ehime 791-0295, Japan; mteraoka@m.ehime-u.ac.jp; 10 Department of Otolaryngology-Head & Neck Surgery, Iwate Medical University, 19-1 Uchimaru, Morioka, Iwate 020-8505, Japan; ymkobaya@iwate-med.ac.jp

**Keywords:** *OTOA*, DFNB22, hearing loss, copy number variations

## Abstract

The *OTOA* gene (Locus: DFNB22) is reported to be one of the causative genes for non-syndromic autosomal recessive hearing loss. The copy number variations (CNVs) identified in this gene are also known to cause hearing loss, but have not been identified in Japanese patients with hearing loss. Furthermore, the clinical features of *OTOA*-associated hearing loss have not yet been clarified. In this study, we performed CNV analyses of a large Japanese hearing loss cohort, and identified CNVs in 234 of 2262 (10.3%, 234/2262) patients with autosomal recessive hearing loss. Among the identified CNVs, *OTOA* gene-related CNVs were the second most frequent (0.6%, 14/2262). Among the 14 cases, 2 individuals carried *OTOA* homozygous deletions, 4 carried heterozygous deletions with single nucleotide variants (SNVs) in another allele. Additionally, 1 individual with homozygous SNVs in the *OTOA* gene was also identified. Finally, we identified 7 probands with *OTOA*-associated hearing loss, so that its prevalence in Japanese patients with autosomal recessive hearing loss was calculated to be 0.3% (7/2262). As novel clinical features identified in this study, the audiometric configurations of patients with *OTOA*-associated hearing loss were found to be mid-frequency. This is the first study focused on the detailed clinical features of hearing loss caused by this gene mutation and/or gene deletion.

## 1. Introduction

Hereditary hearing loss affects approximately one in 500–600 infants in developed countries, and genetic causes account for at least 50% of all childhood hearing loss [1]. Approximately 100 genes have been recognized as causative for sensorineural hearing loss (SNHL) [2]. Next-generation sequencing (NGS) analysis has become a powerful tool for finding variants in many rare genes, and has allowed genetic epidemiology to be clarified [3,4]. We have recently reported a series of studies on various relatively rare genes in the Japanese population, including *POU4F3* [5], *WFS1* [6], *OTOF* [7], and *STRC* [8]. The study was performed as one in a series of findings on specific genes that were published based on the same cohort.

In general, most of the causal mutations in these genes are small insertions/deletions (indels) or single nucleotide variants (SNVs). Recently, copy number variations (CNVs), that is, the alteration through deletion, insertion and/or duplication of more than 1 kbp, involving the genes associated with hearing loss have been observed in several patients with hearing loss (HL) [8,9]. Shearer et al. reported that 143 CNVs were identified in 16 of 89 deafness-associated genes from 686 patients, with the greatest number of CNVs identified in the *STRC* and *OTOA* genes, comprising 73% and 13% of all identified CNVs, respectively [9]. 

The *OTOA* gene (Locus: DNFB22) was first reported as one of the responsible genes for non-syndromic autosomal recessive hearing loss by Zwaenepoel et al. in 2002 [10]. *OTOA* is located on chromosome 16p12.2, and encodes otoancorin, a protein required for limbal attachment of the tectorial membrane, which is important for conditioning proper stimulation of the inner hair cells [11,12].

To date, 27 different variants [9,10,12,13,14,15,16,17,18,19,20,21,22] and 24 long or whole gene deletions [9,13,15,16,19,20,23,24,25] in the *OTOA* gene have been reported to cause SNHL in various ethnic groups, mainly in the Middle-Eastern countries. Although previous papers reported on the SNVs, indels, splicing variants, or CNVs, the detailed clinical characteristics of patients with *OTOA* variants still remain unclear. 

In the present study, we aimed to clarify the prevalence and the clinical characteristics of *OTOA*-associated SNHL by using the NGS platform to identify small variants and CNVs in the *OTOA* gene, and confirmed their existence via direct sequencing or high-resolution array genomic hybridization (aCGH) analysis.

## 2. Materials and Methods 

### 2.1. Subjects

This study was undertaken using data from a total of 2262 Japanese autosomal recessive sensorineural hearing loss (ARSNHL) probands (including sporadic cases) registered from 67 otorhinolaryngology departments in Japan between May 2013 and November 2018. The ages of the probands ranged from 0 to 86 years (mean 21.3 years). To participate in this study, written informed consent was obtained from all patients or the family members of the proband. All procedures were approved by the Shinshu University Ethical Committee as well as the respective ethical committees of the other participating institutions. All methods were in accordance with the Shinshu University Ethical Committee for Human Genetic Research guidelines and regulations. 

This study was conducted in accordance with the Declaration of Helsinki, with the protocol approved by the Ethics Committee of Shinshu University School of Medicine No. 387-4 September 2012 and No. 576-2 May 2017.

### 2.2. Short Variant Analysis Including SNVs, Indels, and Splicing Variants

We developed amplicon libraries, using an Ion AmpliSeq™ Custom Panel (ThermoFisher Scientific, Waltham, MA, USA), for 68 genes previously reported as genetic causes of non-syndromic hearing loss (Supplementary Table S1), and performed emulsion PCR and sequencing, in line with the manufacturer’s instructions. The detailed procedures have been described in our published paper [26]. NGS was performed with an Ion Torrent Personal Genome Machine (PGM) system using an Ion PGM 200 Sequencing Kit and an Ion 318 Chip (ThermoFisher Scientific) or Ion Proton™ system using the Ion PI™ HiQ™ Sequencing 200 Kit and Ion PI™ Chip (ThermoFisher Scientific). We mapped the sequence data against the human genome sequence (build GRCh37/hg19) with a Torrent Mapping Alignment Program. After sequence mapping, the DNA variant regions were stacked with Torrent Variant Caller plug-in software. After variant detection, we analyzed their effects using ANNOVAR software [27]. The variants (missense, nonsense, insertion/deletion and splicing variants) affecting the amino acid sequence were selected from among the identified variants. Variants were further selected as less than 1% of (1) the ExAC [28], (2) gnomAD [29], (3) 3.5KJPN [30] databases, and (4) the 333 in-house Japanese normal hearing controls. We employed direct sequencing to confirm the selected variants.

The pathogenicity of a variant was evaluated based on the criteria of the ACMG (American College of Medical Genetics) standards and guidelines [31]. For missense variants, in particular, functional prediction software, including Sorting Intolerant from Tolerant (SIFT) [32], Polymorphism Phenotyping (PolyPhen2) [33], Likelihood Ratio Test (LRT) [34], Mutation Taster [35], Mutation Assessor [36], Rare Exome Variant Ensemble Learner (REVEL) [37], and Combined Annotation Dependent Depletion (CADD) [38] were used on the ANNOVAR software. We also evaluated the conservation of the variant site in 170 vertebrates from HGMD Professional. [39]. Segregation analysis was performed for each proband and family members (if samples were obtained or available) by direct sequencing. 

### 2.3. Copy Number Variations (CNVs) Analysis

We performed a CNV detection method with Ion AmpliSeq sequencing and multiplex PCR-based targeted genome enrichment. The detailed protocol has been described elsewhere [40]. The read depth data was used for copy number analysis. From the results of the CNVs analysis of the 2262 probands, we picked up 14 patients with *OTOA* gene CNVs.

We designed a custom aCGH for 68 genes previously reported as genetic causes of non-syndromic hearing loss using the Agilent web software (Agilent SureDesign, Agilent Technologies, Santa Clara, CA, USA), with the probes covering specific chromosomal regions of those genes at 150–200 bp intervals as a design-setting on the Agilent 8 × 60 K platform (Agilent Technologies, Santa Clara, CA, USA) [41]. There were 235 probes laid across the *OTOA* region (chr16:21,740,000–21,772,500). We used the same DNA samples as used for the amplicon resequencing, with quality assessment also performed. Five micrograms of genomic DNA were fragmented, and labeled with cyanine-3 for reference DNA samples and cyanine-5 for subjects, and then hybridized. We performed scanning of the array with a G2600D SureScan Microarray Scanner (Agilent Technologies) according to the manufacturer’s recommended protocols, and analyzed scanned aCGH data using CytoGenomics software version 3.0.6.6 (Agilent Technologies).

### 2.4. Clinical Evaluations

Clinical information including the age of onset of SNHL, the result of newborn hearing screening (NHS), pedigree, the presence of subjective progression in SNHL, and episodes of vertigo/dizziness were collected from each proband from a review of the medical charts.

Hearing loss was evaluated using pure-tone audiometry and severity of SNHL was classified by a pure-tone average (PTA) over 500, 1000, 2000 and 4000 Hz. If an individual did not respond to the maximum hearing level at a frequency, 5 dB was added to the maximum hearing level. The severity of HL was classified as follows: mild (PTA: 20–40 dB HL), moderate (41–70 dB HL), severe (71–95 dB HL), and profound (>95 dB HL). Audiometric configuration was categorized into low-frequency, mid-frequency (U-shaped), high-frequency (gently or steeply sloping), or flat based on a previous report [42].

## 3. Results

### 3.1. Identified OTOA Variants and Their Prevalence in Japanese ARSNHL Patients 

**Figure 1:**
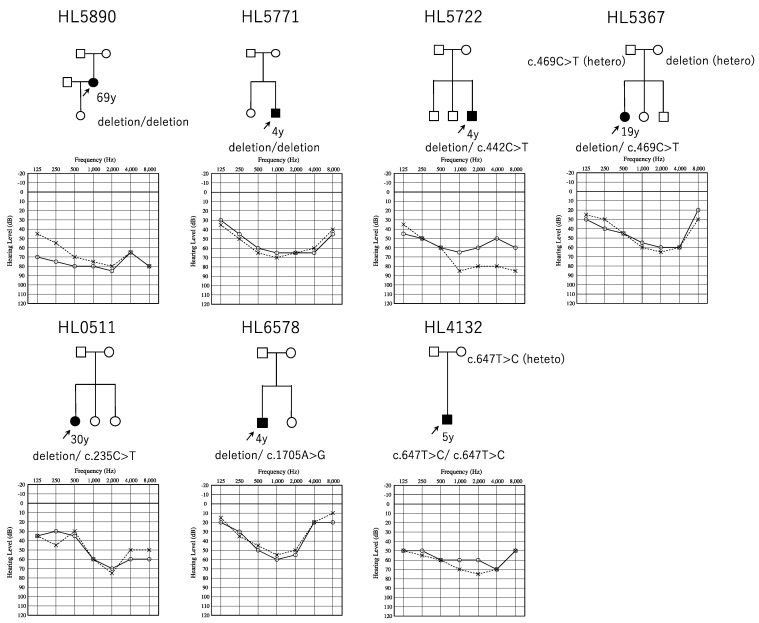
Pedigree and audiograms for each family with *OTOA* variants. Arrows show the probands in each family. The ages indicated in the pedigree represent the time at which the audiogram was obtained. Genetic findings for each individual tested are also noted in the pedigree.

Of 2262 cases, CNVs in the 68 target genes were detected in 234 cases (10.3%, 234/2262). The most frequent gene with CNVs was the *STRC* gene (8.4%, 190/2262), followed by the *OTOA* gene (0.6%, 14/2262). Among the 14 cases with *OTOA* gene CNVs, two carried homozygous deletions, nine carried heterozygous deletions, and three carried three copies (one-copy gain). Among the nine cases with heterozygous deletions in the *OTOA* gene, four cases have possibly disease-causing small variants of the *OTOA* gene in the other allele. Additionally, we identified one case with *OTOA* gene homozygous SNVs. Finally, we identified seven probands with *OTOA*-associated HL in this study (Table 1). Thus, the prevalence of OTOA-associated HL in Japanese ARSNHL patients was calculated to be 0.3% (7/2262). All were sporadic cases, and there were no affected family members (Figure 1). No candidate pathogenic variants in the other 67 deafness genes were detected in these seven individuals. Unfortunately, we could not obtain un-affected sibling samples as shown in Figure 1. Thus, the segregation analysis was not performed for these families.

### 3.2. Confirmation of CNVs and Short Variants, and The Pathogenic Interpretation of These Variants

**Figure 2:**
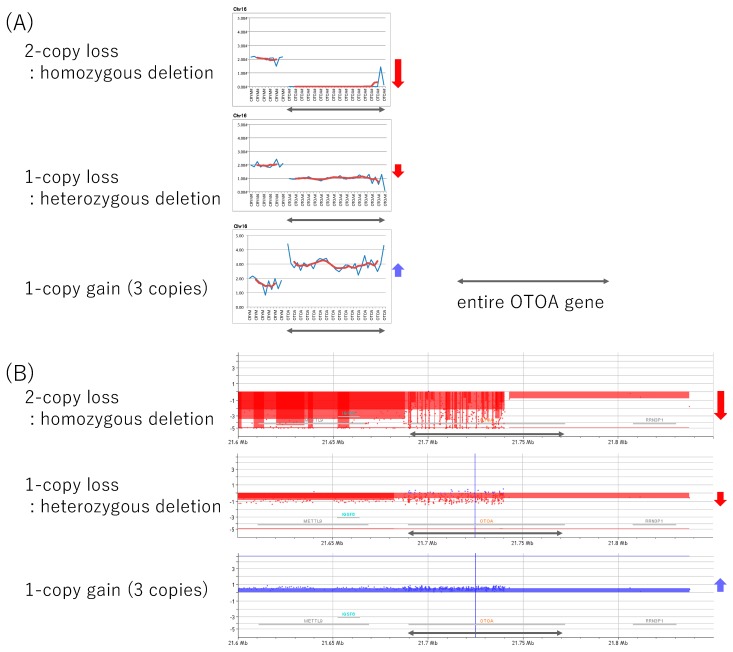
The results of copy number variation (CNV) analysis. (**A**) The results of CNV analysis based on next-generation sequencing (NGS) read depth data for patients with two-copy loss (homozygous deletion), one-copy loss (heterozygous deletion), or one-copy gain (three copies) in the *OTOA* gene identified in the present study. (**B**) The results of aCGH analysis for the same patients. Black arrows indicate the *OTOA* region. Red arrows indicate deletions, and blue arrows indicate duplications.

In this study, we detected CNVs by using NGS read data as a first screening step followed by confirmation with aCGH. We performed aCGH analysis to confirm the CNVs for five individuals. Two of them (HL5890 and HL5771) carried homozygous deletions in the *OTOA* gene and three (HL5722, HL5367, and HL6578) carried heterozygous deletions. All cases had entire *OTOA* gene deletions, and the aCGH results were consistent with the NGS-based analysis results. Furthermore, deletions in all cases included the *METTL9* and *IGSF6* genes, which are located upstream of the *OTOA* gene. Figure 2 shows the results of NGS analysis and aCGH analysis in these cases. We also performed aCGH analysis for a case with three copies as a technical confirmation, and the results were consistent with the NGS analysis results. Therefore, we believe that CNV analysis using NGS data is reliable, even for heterozygous deletions, homozygous deletions, and one-copy gains in the *OTOA* gene. Unfortunately, the total amount of DNA available for HL0511 was not sufficient for aCGH analysis, so we did not perform aCGH analysis for this patient. 

All five single nucleotide variants (c.235C>T, c.442C>T, c.469C>T, c.1705A>G, and c.647T>C) identified in this study were evaluated according to the ACMG standards and guidelines [31]. All variants were novel, and were not observed or observed in very low frequency in the control population database (PM2) (Table 2). One mutation (c.442C>T) was categorized as a “likely pathogenic” variant as this variant is a nonsense variant (p.(Asp148^*^)) leading to the stop codon (PVS1). Three missense variants (c.235C>T, c.469C>T, and c.1705A>G) detected in *trans* with a pathogenic (whole gene deletion) variant (PM3) were categorized as being of “uncertain significance”. The remaining missense variant identified as homozygous (c.647T>C) was also categorized as of “uncertain significance”. All four missense variants were predicted as deleterious and have high CADD scores.

### 3.3. Clinical Features of OTOA-Associated SNHL Patients

Table 1 summarizes the clinical findings of the seven affected individuals identified in this study. The age of onset of HL ranged from congenital to childhood. All congenital cases were identified through NHS, but the other childhood onset cases did not receive NHS. Most of the cases have bilateral symmetrical SNHL (Figure 1), and the severity of HL ranged from moderate to severe. Interestingly, most cases showed mid-frequency HL. Based on the audiometric configuration classification criteria previously reported, mid-frequency HL was observed in nine ears, flat type in three ears, and high-frequency HL in two ears. Progression of HL was noticed, based on the medical charts, for three (all adults: HL5890, HL0511, and HL5367) of the seven individuals. Serial audiograms could be obtained from one individual (HL5367), and the averaged hearing threshold (PTA) was observed to have slowly deteriorated from 41.25 dB at 4 years old to 55 dB at 19 years old. Vertigo/dizziness is rare among patients with *OTOA*-associated HL, and only one individual (HL5890) was found to have episodes of vertigo.

## 4. Discussion 

In our cohort of 2262 Japanese ARSNHL patients, we identified seven probands with *OTOA*-associated HL, including two cases with homozygous deletions, four cases with heterozygous deletions in *trans* to a SNVs, and one case with homozygous SNVs. The frequency of *OTOA*-associated HL in Japanese ARSNHL patients was calculated to be 0.3% (7/2262). In a previous report analyzing a larger number of patients, Shearer et al. identified five probands with *OTOA*-associated HL among 686 SNHL patients from American probands, so that the frequency of *OTOA*-associated HL was calculated to be 0.7% among all SNHL patients (5/686) [9]. Sloan-Heggen et al. identified eight probands with *OTOA*-associated HL among 1119 unrelated SNHL patients from various ethnic populations (0.7%) [16] and also identified six *OTOA*-associated HL cases among 302 Iranian patients (2.0%) [13]. Our results were comparable with the studies on both the American patients and various ethnic populations, but noticeably lower than that on the Iranian patients. These differences may reflect differences in the ratio of consanguineous patients among each cohort. 

To elucidate the prevalence of *OTOA* CNVs in the normal hearing population, we also performed NGS analysis for 152 normal hearing controls (data not shown). The controls were aged from 20–30 years, and pure-tone audiometry was performed for each control, showing normal hearing. Among the 152 controls, none carried a copy number loss of the *OTOA* gene, but one case carried three copies of the *OTOA* gene. It was unclear whether the one-copy gain of the *OTOA* gene was pathogenic or neutral (no impact on phenotype). However, the identification of a one-copy gain of the *OTOA* gene from a control case, suggests that this one-copy gain of the *OTOA* gene was not associated with any phenotypes. Therefore, the CNVs of *OTOA* were rare in Japanese control population.

For all *OTOA* gene CNVs identified in this study, the aCGH results showed that the whole *OTOA* gene as well as whole *METTL9* and *IGSF6* genes were deleted or duplicated. In the previous three reports analyzing the deletion region in detail [23,24,43], all cases carried a whole *OTOA*, *METTL9* and *IGSF6* gene deletion as in this study. One plausible reason for relatively large number of CNVs observed in this area and same types of deletion including *OTOA*, *METTL9,* and *IGSF6* were observed even in different ethnic population, is the segmental duplications of the region in chromosome 16p12.2. There is a highly homologous sequences before and after chr16p12.2, including the *OTOA*, *METTL9,* and *IGSF6* genes [23,43,44,45]. Further, these segmental duplication increased mis-homologous recombination in this region, and may act as a hotspot for CNVs. As a result of this mis-homologous recombination, the similar CNVs in this area (including the *OTOA*, *METTL9,* and *IGSF6* genes) may be commonly observed in many ethnic populations.

The *OTOA* gene has a pseudogene located 820Kb downstream, which has a high sequence similarity and 99% or more homology in the exon 20–28 region of the *OTOA* gene [23]. Therefore, the mapping quality of this region was degraded and SNV detection in this region is challenging when using short-read NGS [46]. Except for one variant (c.2960_2961delAT), all variants identified in this study and previous studies were located in exon 3-19 (summarized in Table 3).

In this study, we identified nine cases with one-copy loss of the *OTOA* gene. Among these nine cases, four cases carried one-copy loss of the OTOA gene with candidate SNVs in the *trans* allele; however, five cases carried only one-copy loss of the *OTOA* gene. Shearer et al. also reported five cases among 686 cases that carried one-copy loss of the *OTOA* gene without any other SNVs in the *OTOA* gene [4]. Among these cases, there might be some cases with SNVs in the exon 20–28 region that cause *OTOA*-associated HL. To confirm these cases, newer technologies such as long-read NGS are required.

In this study, we used aCGH to confirm the CNVs identified from NGS results. Array CGH has been the gold standard for copy number analysis, but it is time-consuming and costly. Thus, now we employ NGS as the standard CNVs analysis method as it is possible to detect the SNVs and CNVs in one experiment [15]. In addition, we are currently trying to establish a social health insurance-based platform using NGS as standard CNV detection method as it is possible to detect SNVs and CNVs at the same time and it is more cost- and time-effective.

The severity of the *OTOA*-associated HL varied from moderate to severe, but most of the cases showed moderate HL (86%, 6/7 individuals) in this study. Also in previous reports, the severity of HL varied significantly from mild to profound (summarized in Table 4). Even in cases of homozygous *OTOA* gene deletions, significant differences were observed in the severity of HL. These differences in the severity of HL may be due to other environmental or genetic factors including aging. The progress of HL in patients with *OTOA*-associated HL has not been specifically described in previous reports. In the present study, three adult cases noticed progression of HL, and the progression was confirmed by serial audiograms in one patient in whom the averaged hearing threshold (PTA) was slowly deteriorated from 41.25 dB at 4 years old to 55 dB at 19 years old. From these observations, progressive HL appears to be a common trend in *OTOA*-associated HL. With regard to the age of onset, three cases showed congenital HL and others showed prelingual to childhood onset in this study. In previous reports, the age of onset was pre-childhood in most cases, but two cases of adult onset were reported [9,16]. All three cases with congenital HL identified in this study were identified through NHS screening. Thus, we estimated that most cases of *OTOA*-associated HL may be congenital and could be identified through NHS screening. However, in cases not undergoing such screening, the HL was mild to moderate and progressed slowly, and was identified in childhood or later.

It is noteworthy that mid-frequency HL was most commonly observed in individuals with *OTOA* variants in this study. In addition, flat HL and high-frequency HL were also observed in some cases. In previous reports, only Alkowari et al. have provided detailed audiograms of the three cases from one family with homozygous *OTOA* deletions, and the audiometric configurations of these patients were mid-frequency HL [25]. Interestingly, otoancorin, encoded by the *OTOA* gene, is a protein that acts as a glycosylphosphatidylinositol (GPI) anchorage, and is important for limbal attachment of the tectorial membrane (TM) [11,12]. The *TECTA* gene (Locus: DFNA8/12) encoding α-tectorin, a major non-collagenous glycoprotein of TM, which is expressed in the spiral limbus during TM development [10,11], is also known as a genetic cause of mid-frequency HL [47,48,49]. The similarities between the clinical characteristics of HL in patients with *OTOA* and *TECTA* gene mutations reflect the mechanism of deafness caused by TM impairment. The results of this study will be useful for the selection of more appropriate treatment for patients as well as the further understanding of the disease-causing mechanisms of *OTOA*-associated HL.

## 5. Conclusions

Here, we presented the detailed clinical characteristics of the seven patients with *OTOA*-associated HL identified from 2262 unrelated Japanese ARNSHL patients. The prevalence of *OTOA*-associated HL in Japanese ARSNHL patients was calculated to be 0.3%. This is the first report of HL caused by this gene mutation in Japanese patients with HL. The remarkable clinical characteristics of the patients with *OTOA* variants was congenital or early onset, progressive, mid-frequency HL.

## Figures and Tables

**Table 1:** Summary of the clinical features and identified variants of individuals with *OTOA* variants in this study.

							Newborn	Average		Audiometric	Hearing	
							Hearing	Hearing Level	Age at	Configuration	Loss	Vertigo/
ID	Zygosity	Allele #1		Allele #2		Onset	Screening R/L	R/L (dB)	Audiogram	R/L	Progression	Dizziness
HL5771	homo	whole gene deletion		whole gene deletion		3y	N/A	58.75/62.5	4y	MF/MF		-
HL5890	homo	whole gene deletion		whole gene deletion		childhood	N/A	77.5/72.5	69y	Flat/MF	progressive	+
HL0511	compound hetero	whole gene deletion		c.235C>T	p.(Arg79Trp)	7y	N/A	56.25/55	30y	HF/MF	progressive	-
HL5722	compound hetero	whole gene deletion		c.442C>T	p.(Arg148*)	0m	refer/refer	58.75/76.25	7y	Flat/HF		-
HL5367	compound hetero	whole gene deletion		c.469C>T	p.(Arg157Cys)	5y	N/A	55/57.5	19y	MF/MF	progressive	-
HL6578	compound hetero	whole gene deletion		c.1705A>G	p.(Lys569Glu)	0m	refer/refer	46.25/42.5	4y	MF/MF		-
HL4132	homo	c.647T>C	p.(Phe216Ser)	c.647T>C	p.(Phe216Ser)	0m	refer/refer	62.5/68.75	5y	Flat/MF		-

All variants were indicated in NM_144672. y: year(s), m: month(s), N/A: not applicable (not received NHS), HF: high-frequency sensorineural hearing loss, MF: mid-frequency sensorineural hearing loss.

**Table 2:** Possible causative variant identified in this study.

		Prediction Score							Allele Frequency in Controls				
	Amino												
Nucleotide	Acid		PolyPhen		Mut_	Mut_						ACMG	
Changes	Change	SIFT *	2_HVAR *	LRT *	Taster *	Assessor *	REVEL *	Cadd	Exac	Gnomad	3.5kJPN	Guidelines	
c.235C>T	p.(Arg79Trp)	D(0.4)	B(0.166)	N(0.132)	N(0.09)	M(0.552)	0.21	23.6	0.00000824	0.00000812	N/A	Uncertain Significance	PM2,PM3
c.442C>T	p.(Arg148*)	-	-	N(0.225)	A(0.81)	-	-	35	0.0000247	0.0000163	N/A	Likely Pathogenic	PVS1, PM2
c.469C>T	p.(Arg157Cys)	D(0.912)	D(0.916)	D(0.629)	D(0.548)	M(0.752)	0.285	34	0.0000165	0.0000203	N/A	Uncertain Significance	PM2,PM3
c.1705A>G	p.(Lys569Glu)	D(0.427)	D(0.875)	D(0.629)	D(0.441)	M(0.567)	0.598	31	N/A	N/A	N/A	Uncertain Significance	PM2,PM3
c.647T>C	p.(Phe 216Ser)	D(0.721)	D(0.764)	D(0.629)	D(0.412)	M(0.741)	0.326	24.3	N/A	N/A	N/A	Uncertain Significance	PM2

* The Prediction Score of each algorithm included in the ANNOVAR software was converted from the original scoring system. A score closer to 1.0 indicated the variant was predicted to be more damaging. A, disease causing automatic (Mutation Taster); B, benign (PolyPhen2_HVAR); D, deleterious (SIFT, LRT), probably damaging (PolyPhen2), or disease causing (Mutation Taster); M, medium (Mutation Assessor); N, Neutral (LRT). PVS: evidence of Pathogenicity—Very Strong, PM: evidence of Pathogenicity—Moderate.

**Table 3:** Summary of variants identified in this and previous studies (NM_144672).

			Allele Frequency		Prediction Score							
Nucleotide	Amino Acid			GnomAD		Polyphen2		Mut	Mut			
Change	Change	Exon	Exac03	Exome	Sift *	_Hvar *	LRT *	Taster *	Assessor *	Revel *	CADD	Reference
missense/nonsense variant												
c.131T>C	p.(Ile44Thr)	3	0.0000494	0.0000731	D	P	D	D	M	N/A	23.8	Christina M. Sloan-Heggen, 2016 [16]
c.235C>T	p.(Arg79Trp)	5	0.00000824	0.00000812	D(0.4)	B(0.166)	N(0.132)	N(0.09)	M(0.552)	0.21	23.6	this study
c.313A>T	p.(Lys105*)	6	N/A	N/A	-	-	-	-	-	-	-	Christina M. Sloan-Heggen, 2016 [16]
c.442C>T	p.(Arg148*)	7	0.0000247	0.0000163	-	-	N(0.225)	A(0.81)	-	-	35	this study
c.446C>A	p.(Ala149Asp)	7	0.000016	N/A	D	D	N	P	-	-	28.8	Shearer, 2014 [9]
c.469C>T	p.(Arg157Cys)	7	0.0000165	0.0000203	D(0.912)	D(0.916)	D(0.629)	D(0.548)	M(0.752)	0.285	34	this study
c.647T>C	p.(Phe216Ser)	8	N/A	N/A	D(0.721)	D(0.764)	D(0.629)	D(0.412)	M(0.741)	0.326	24.3	this study
c.878A>G	p.(Gln293Arg)	10	N/A	N/A	D	P	D	D	M	-	24.2	L. He, 2018 [17]
c.1025A>T	p.(Asp342Val)	11	N/A	N/A	D(0.784)	D(0.719)	N(0.388)	D(0.81)	M(0.552)	0.453	26.7	Walsh, 2006 [18]
c.1249C>T	p.(Leu417Phe)	12	0.0000165	0.0000163	D	P	D	D	M	-	28.6	Tsai, 2013 [19]
c.1282G>T	p.(Val428Phe)	12	N/A	N/A	D	P	N	P	L	-	24.7	Cabanillas, 2018 [20]
c.1352G>A	p.(Gly451Asp)	13	0.00000824	0.00000407	D(0.912)	D(0.971)	D(0.439)	D(0.524)	M(0.567)	0.768	24.8	K Lee, 2013 [21]
c.1705A>G	p.(Lys569Glu)	16	N/A	N/A	D(0.427)	D(0.875)	D(0.629)	D(0.441)	M(0.567)	0.598	31	this study
c.1728T>G	p.(Ile576Met)	16	0.000033	0.0000284	D	P	D	D	M	-	23.8	Christina M. Sloan-Heggen, 2016 [16]
c.1865T>A	p.(Leu622His)	17	0.000008	N/A	D	P	D	D	-	-	29.1	P Fontana, 2017 [15]
c.1807G>T	p.(Val603Phe)	16	N/A	0.00000406	T	P	N	D	M	-	26.6	Ammar-Khodja, 2015 [22]; Christina M. Sloan-Heggen, 2016 [16]
c.1814G>C	p.(Cys605Ser)	17	N/A	N/A	T	P	D	D	M	-	26.8	Christina M. Sloan-Heggen, 2016 [16]
c.1879C>T	p.(Pro627Ser)	17	0.000033	0.0000366	D(0.496)	D(0.916)	D(0.629)	D(0.548)	M(0.567)	0.446	31	K Lee, 2013 [21]; Christina M. Sloan-Heggen, 2015 [13]
c.1939G > C	p.(Gly647Arg)	18	N/A	0.0000122	T(0.363)	P(0.604)	D(0.629)	D(0.478)	M(0.567)	0.813	23.6	Christina M. Sloan-Heggen, 2015 [13]
c.2201A>G	p.(Gln734Arg)	19	0.00000824	0.00000407	T(0.330)	B(0.339)	N(0.229)	D(0.330)	M(0.723)	0.079	8.163	Christina M. Sloan-Heggen, 2015 [13]
splicing variant												
c.151+1G>A			N/A	N/A	-	-	-	D(0.81)	-	-	26.3	Christina M. Sloan-Heggen, 2015 [13]
c.1320+2T>C			N/A	N/A	-	-	-	D(0.81)	-	-	24.2	Zwaenepoel, 2002 [10]
c.1320+5G>C			N/A	0.00001	-	-	-	D	-	-	21.7	Bong Jik Kim, 2019 [12]
c.2208−1G>A			0.000036	N/A	-	-	-	D(0.81)	-	-	22.4	Christina M. Sloan-Heggen, 2015 [13]
small deletion												
c.827delT	p.(Ile276fs)	9	0.000025	N/A	-	-	-	N/A	-	-	35	Shearer, 2014 [9]; Christina M. Sloan-Heggen, 2016 [16]; Sommen, 2016 [14]
c.1765delC	p.(Gln589fs)	17	0.000025	N/A	-	-	-	D	-	-	28.5	Bong Jik Kim, 2019 [12]
c.2960_2961delAT	p.(His987fs)	25	0.000094	N/A	-	-	-	N/A	-	-	25.3	Sommen, 2016 [14]

All variants were indicated in NM_144672. * The Prediction Score of each algorithm included in the ANNOVAR software was converted from the original scoring system. A score closer to 1.0 indicated the mutation was more damaging, and that closer to 0 indicated it was more tolerant. A disease causing automatic (Mutation Taster); B, benign (PolyPhen2); D, deleterious (SIFT, LRT), probably damaging (PolyPhen2), or disease causing (Mutation Taster); L, low (Mutation Assessor); M, medium (Mutation Assessor); N, Neutral (LRT), polymorphism (Mutation Taster); P, possibly damaging (PolyPhen2), polymorphism automatic (Mutation Taster); T, Tolerated (SIFT).

**Table 4:** Summary of clinical features associated with *OTOA* variants from this and previous studies.

Hereditary	Onset	Average hearing level	Zygosity	Allele #1		Allele #2		Reference
AR/Spo	3y	moderate	homo	whole gene deletion		whole gene deletion		this study
AR/Spo	childhood	severe	homo	whole gene deletion		whole gene deletion		this study
AR	prelingual	N/A	homo	whole gene deletion		whole gene deletion		Shahin, 2010 [23]
AR	N/A	mild to moderate	homo	whole gene deletion		whole gene deletion		Bademci, 2014 [24]
AR	0−10y	moderate to severe	homo	Whole gene deletion		whole gene deletion		Shearer, 2014 [9]
N/A	21−30y	N/A	homo	whole gene deletion		whole gene deletion		Shearer, 2014 [9]
AR	prelingual	moderate to severe	homo	whole gene deletion		whole gene deletion		Christina M. Sloan-Heggen, 2015 [13]
N/A	N/A	N/A	homo	whole gene deletion		whole gene deletion		Christina M. Sloan-Heggen, 2016 [16]
AD	adult	severe to profound	homo	whole gene deletion		whole gene deletion		Christina M. Sloan-Heggen, 2016 [16]
Spo	congenital	severe to profound	homo	whole gene deletion		whole gene deletion		Christina M. Sloan-Heggen, 2016 [16]
AR	1−13y	severe	homo	58000bp deletion		58000bp deletion		Alkowari, 2017 [25]
AR	prelingual	severe	homo	c.151+1G>A		c.151+1G>A		Christina M. Sloan-Heggen, 2015 [13]
AR/Spo	0m	moderate	homo	c.647T>C	p.(Phe216Ser)	c.647T>C	p.(Phe216Ser)	this study
AR	prelingual	moderate to severe	homo	c.1025A>T	p.(Asp342val)	c.1025A>T	p.(Asp342val)	Walsh, 2006 [18]
AR	prelingual	moderate to severe	homo	c1320+2T>C		c.1320+2T>C		Zwaenepoel, 2002 [10]
AR	prelingual	severe	homo	c.1352G>A	p.(Gly451Asp)	c.1352G>A	p.(Gly451Asp)	K Lee, 2013 [21]
AR	prelingual	severe to profound	homo	c.1807G>T	p.(Val603Phe)	c.1807G>T	p.(Val603Phe)	Ammar-Khodja, 2015 [22]
AR	prelingual	severe	homo	c.1879C>T	p.(Pro627Ser)	c.1879C>T	p.(Pro627Ser)	K Lee, 2013 [21]
AR	prelingual	moderate to severe	homo	c.1879C>T	p.(Pro627Ser)	c.1879C>T	p.(Pro627Ser)	Christina M. Sloan-Heggen, 2015 [13]
AR	prelingual	moderate to severe	homo	c.1939G C	p.(Gly647Arg)	c.1939G>C	p.(Gly647Arg)	Christina M. Sloan-Heggen, 2015 [13]
AR	prelingual	moderately severe to profound	homo	c.2201A>G	p.(Gln734Arg)	c.2201A>G	p.(Gln734Arg)	Christina M. Sloan-Heggen, 2015 [13]
AR/Spo	7y	moderate	compound hetero	whole gene deletion		c.235C>T	p.(Arg79Trp)	this study
N/A	0−10y	N/A	compound hetero	whole gene deletion		c.446C>A	p.(Ala149Asp)	Shearer, 2014 [9]
AR/Spo	5y	moderate	compound hetero	whole gene deletion		c.469C>T	p.(Arg157Cys)	this study
N/A	0−10y	N/A	compound hetero	whole gene deletion		c.827delT	p.(Ile276fs)	Shearer, 2014 [9]
Spo	congenital	N/A	compound hetero	whole gene deletion		c.827delT	p.(Ile276fs)	Christina M. Sloan-Heggen, 2016 [16]
AR	childhood	N/A	compound hetero	whole gene deletion		c.1282G>T	p.(Val428Phe)	Cabanillas, 2018 [20]
AD	congenital	N/A	compound hetero	whole gene deletion		c.1728T>G	p.(Ile576Met)	Christina M. Sloan-Heggen, 2016 [16]
AR/Spo	congenital	moderate	compound hetero	whole gene deletion		c.1705A>G	p.(Lys569Glu)	this study
Spo	childhood	severe to profound	compound hetero	whole gene deletion		c.1807G>T	p.(Val603Phe)	Christina M. Sloan-Heggen, 2016 [16]
Spo	congenital	mild to moderate	compound hetero	whole gene deletion		c.1814G>C	p.(Cys605Ser)	Christina M. Sloan-Heggen, 2016 [16]
AR	prelingual	severe	compound hetero	whole gene deletion		c.1865T>A	p.(Leu622His)	P Fontana, 2017 [15]
N/A	N/A	N/A	compound hetero	multi exon deletion		c.1249C>T	p.(Leu417Phe)	Tsai, 2013 [19]t
AR/Spo	0m	moderate	compound hetero	deletion		c.442C>T	p.(Arg148*)	this study
AR	prelingual	N/A	compound hetero	deletion		c.2960_2961delAT	p.His987fs	Sommen, 2016 [14]
Spo	before 6 years	moderate	compound hetero	micro deletion		c.878A>G	p.(Gln293Arg)	L. He, 2018 [17]
Spo	congenital	mild to moderate	compound hetero	c.131T>C	p.(Ile44Thr)	c.313A>T	p.(Lys105*)	Christina M. Sloan-Heggen, 2016 [16]
AR	prelingual	N/A	compound hetero	c.827delT	p.(Ile276fs)	c.2960_2961delAT	p.(His987fs)	Sommen, 2016 [14]
AR	congenital	moderate	compound hetero	c.1320+5G>C		c.1765delC	p.(Gln589fs)	Bong Jik Kim, 2019 [12]

AD: autosomal dominant. AR: autosomal recessive. Spo: sporadic. N/A: not available. y: year(s), m: month(s).

## Data Availability

The sequencing data are available in the DDBJ databank of Japan (Accession number: JGAS00000000200).

## Supplementary Materials

The following are available online at https://www.mdpi.com/2073-4425/10/9/715/s1, Table S1: 68 deafness-causative genes.
